# Rho Small GTPase Family in Androgen-Regulated Prostate Cancer Progression and Metastasis

**DOI:** 10.3390/cancers17223680

**Published:** 2025-11-17

**Authors:** Dontrel William Spencer Hairston, Maria Mudryj, Paramita Mitra Ghosh

**Affiliations:** 1 Research Service, VA Northern California Health Care System, Mather, CA 95655, USA; 2 Department of Urologic Surgery, University of California Davis, Sacramento, CA 95817, USA; 3 Department of Medical Microbiology and Immunology, University of California Davis, Davis, CA 95616, USA; 4 Department of Biochemistry and Molecular Medicine, University of California Davis, Sacramento, CA 95817, USA

**Keywords:** Rho small GTPases, Rho, Rac, Cdc42, androgen receptor, RhoGEF, RhoGAP, RhoGDI

## Abstract

**Background/Objectives**: Rho small GTPases (RSG), which regulates metastasis, constitute eight subfamilies—“classical” Rho, Rac, cdc42, and “atypical” Rif, Rnd, Wrch, RhoH, and RhoBTB. Their downstream signaling requires switching between GTP-bound active and GDP-bound inactive forms. Classical RSGs, but not atypical RSGs, require regulation by guanine nucleotide exchange factors (GEF), GTPase-activating proteins (GAP) and guanine nucleotide dissociation inhibitors (GDI) to achieve this switch. The objective of this review is to summarize the roles of RSGs in metastatic prostate cancer (mPCa) and their interaction with the androgen receptor (AR), which regulates this disease. **Methods**: We summarize the literature that describes the role of RSGs in mPCa, and their interaction with the AR. **Results**: Classical RSGs mostly promote metastasis (except RhoB), whereas atypical RSGs, with exceptions, mostly prevent it. Their role, however, is context-dependent—e.g., RhoB is tumor-suppressive in AR-null PCa but oncogenic in AR-positive tumors. The AR modulates RSG expression transcriptionally, but also affects their function through modulation of GEFs, GAPs, and GDIs. In turn, RSGs also regulate AR transcriptional activity. Interestingly, RSGs and the AR have non-genomic interactions via membrane-localized AR (mAR) not affected by AR inhibitors. **Conclusions**: Drugs that target RSGs are needed along with AR inhibitors to prevent mPCa progression.

## Simple Summary

While local and regional prostate cancer (PCa) maintains a nearly 100% 5-year overall survival, metastatic PCa (mPCa) reduces 5-year overall survival to 38%. Since PCa is strongly regulated by the androgen receptor (AR), initial treatment for mPCa involves drugs that inhibit the AR pathway. However, resistance to these treatments eventually ensue, resulting in castration-resistant PCa (CRPC). Understanding the biology behind mPCa progression is therefore necessary to identify additional treatments that may prevent this progression. One of the most studied pathways that regulate metastasis is the 20-member Rho small GTPase (RSG) family that regulate migration and invasion and activate downstream targets by switching from a guanosine triphosphate (GTP)-bound active form into a guanosine diphosphate (GDP)-bound inactive form. In this systematic review, we explore the role of the Rho GTPases in mPCa and the interaction between classical Rho GTPases with the AR that regulates the progression of this disease.

## 1. Introduction

Prostate cancer (PCa) is associated with uncontrolled growth and proliferation in cells of the prostate gland [1,2]. The prostate produces prostate-specific antigen (PSA), a protease that is commonly utilized to evaluate prostate health. PSA expression is modulated by the transcriptional activity of the ligand-activated androgen receptor (AR), a steroid hormone receptor which regulates almost all aspects of prostate health and is implicated in PCa diagnosis and prognosis. Among all cancer types in the U.S., PCa is second in estimated new cases (14.7%) and fifth in estimated deaths (5.7%) [3]. While local and regional PCa maintain a >99% 5-year survival rate, progression to distant metastasis (mPCa) reduces this to 38% [1,3]. This systematic review will therefore focus on a pathway involved in modulating metastasis in PCa.

The Rho (Ras homologous) family of small GTPases (RSG) play a crucial role in PCa metastasis by regulating cell migration, invasion, and epithelial–mesenchymal transition (EMT). They fall under the umbrella of the Ras superfamily and are activated when GTP-bound [4]. The human Rho GTPase family consists of 20 members categorized into eight subfamilies: Rho, Rac, Cdc42, Rif, Rnd, Wrch, RhoH, and RhoBTB (Table 1). These subfamilies are further classified as “classical” members that switch between active GTP-bound and inactive GDP-bound states and “atypical” members that are mostly active.

Three other members, RhoBTB3, Miro1, and Miro2, that were once thought to be members of this family are no longer considered to be RSGs as they substantially differ from the other members. For example, Miro contains two EF-hand (EFH) motifs with another GTPase domain and is missing the Rho insert region that is found in all RSGs. RhoBTB and Miro are absent of post-translational modifications and a -CAAX motif. Their roles in PCa pathology and progression are listed below (Table 2) [5]. This systematic review will therefore investigate the overall role of the 20 member RSG family in PCa metastasis, identifying members and regulators that may be involved in migration and invasion of PCa cells.

## 2. Mechanisms Implicated in PCa Progression

### 2.1. Structure of the AR and Its Role in PCa Initiation and Development

**Figure 1:**
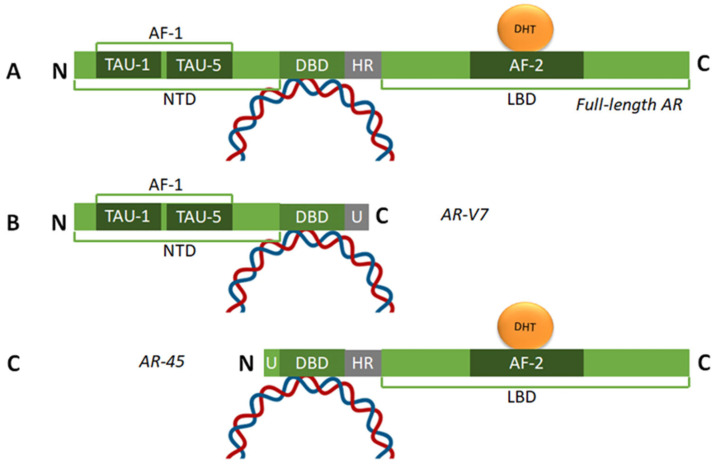
Structure of the androgen receptor (AR). (**A**). AR gene transcription and translation lead to AR protein expression which is typically composed of an E1-derived N-terminal domain (NTD) containing activation factor 1 (AF-1) region, followed by an E2- and E3-derived DNA-binding domain (DBD), an E4-derived hinge region (HR), and an E4 thru E8-derived ligand-binding domain (LBD) containing activation factor 2 (AF-2) region. Tau-1 and Tau-5 are regions within AF-1 that play major roles in AR transactivation. (**B**) Constitutively active AR-V7 is truncated after E3 and contain carboxy-terminal (C-terminal) amino acids unique (U) to the C-terminal end (CE) where the truncation took place (CE AAs). (**C**) In contrast, AR45 lacks the NTD and can still bind ligands.

PCa growth and progression are initially reliant on androgen binding to the AR, a ligand-dependent X-linked 90 kb, 110 kDa receptor comprising ~919 amino acids encoded by eight exons [6,7]. The AR is highly expressed in male reproductive organs like the prostate, epididymis, and testes and also expressed at lower levels in non-reproductive organs such as the brain, muscle, hair follicles, and adipose tissue in both males and females [8]. Distinct functional motifs on the AR include the N-terminal domain (NTD) encoded by exon 1 and containing an AF-1 transactivation domain, a DNA-binding domain (DBD) encoded by exons 2 and 3, a hinge region encoded by the 5′ region of exon 4, and a C-terminal ligand-binding domain (LBD) encoded by exons 4 thru 8 containing the AF-2 transactivation domain [6,7] (Figure 1). Interaction between the AR N- and C-termini is required for AR transcriptional activity.

Testosterone (T) is the most common androgen in males in most tissues [9], whereas in the prostate, dihydrotestosterone (DHT) is more prevalent. DHT binds to the AR with a binding affinity much higher than that of T [10]. Leutenizing hormone-releasing hormone (LHRH), produced by the hypothalamus, controls T production in testicular tissue [10]. T and DHT are considered primary AR ligands; T is converted to DHT by the enzyme 5α-reductase, although adrenal androgen such as dehydroepiandrosterone (DHEA, the most common androgen in females) also binds AR [1,6].

Due to the heavy reliance of hormone-sensitive PCa (HSPC) on the AR, androgen deprivation therapy (ADT) is used in the treatment of stage III PCa and beyond, with or without adjuvant radiation therapy [11]. LHRH agonists such as leuprolide and goserelin acetate are effective in reducing T and DHT production and inhibiting PCa proliferation [12]. Adrenal glands may also produce DHT from DHEA, thereby bypassing LHRH-dependent T production [13]. To prevent these so-called “backdoor androgen pathways”, additional inhibitors of DHT production, such as abiraterone acetate (ABI), have been developed [14]. Several other AR signaling inhibitors (ARSI) used in high-risk patients with localized PCa in combination with ADT include non-steroidal anti-androgens such as flutamide, bicalutamide, and nilutamide, and AR inhibitors such as enzalutamide, apalutamide, and darolutamide that directly interact with the AR-LBD to ultimately reduce AR-mediated signaling [1,6,15,16].

**Figure 2:**
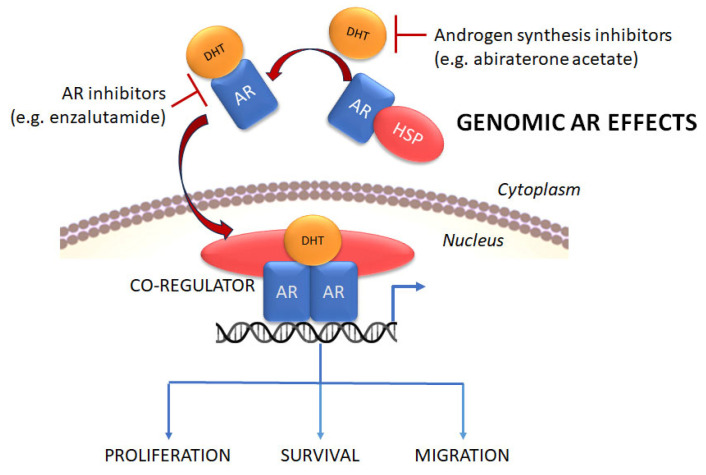
Genomic effects of the androgen receptor. The AR mediates the genomic effects of androgens by acting as a ligand-dependent transcription factor that binds to DNA, regulating the expression of target genes to influence processes such as male sexual differentiation, muscle development, and prostate function. This binding activates or represses gene transcription through interaction with cofactors and chromatin remodeling complexes, leading to changes in protein synthesis that control cell behavior and physiology. In the cytoplasm, the AR binds to, and is inactivated by, chaperone HSPs. Upon ligand binding, the AR undergoes conformational changes, detaches from the HSPs, and translocates to the nucleus (indicated by arrows). In the nucleus, the AR binds to AREs located in the regulatory regions of target genes. This recruits a variety of cofactors, which can either activate or repress the transcription of the associated genes.

The AR in the inactive state is localized to the cytoplasm, complexed with heat shock proteins (HSP), but upon ligand binding at the LBD, dissociates from the HSPs, and translocates into the nucleus [10] (Figure 2). Here, the AR binds to androgen-responsive elements (ARE) in the target genes to modulate expression of AR-responsive transcriptional targets [10]. Regulation of AR signaling is important in both the activation and repression of transcription. In non-tumor prostate, low androgen concentrations initiate transcription and promote growth, whereas high androgen levels trend toward growth inhibition [17]; however, in PCa tissue, where the AR or its transcriptional co-regulators may be overexpressed or mutated, high levels of androgens promote both growth and migration due to altered transcriptional programs [18]. Thus, the normal prostate, as well as early and/or untreated PCa, relies heavily on ligand-dependent AR.

### 2.2. The Role of the AR in PCa Metastasis

**Figure 3:**
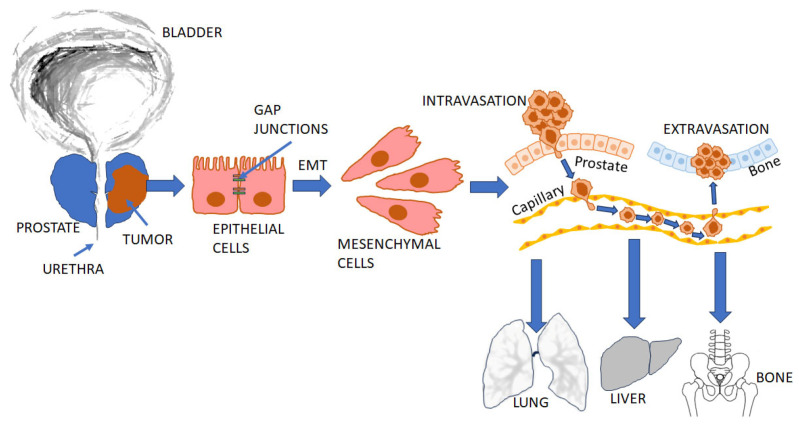
Mechanism of prostate cancer metastasis to distant sites. The prostate gland encircles the urethra below the bladder. Growth from multiple focal points lead to tissue disorganization and a reduction in the size of ductal structures that precede tissue invasion and metastasis. After tumor cells experience intravasation, travel through the circulatory system, and extravasate and exit the bloodstream at a favorable tumor site, PCa will seed and proliferate at sites within distant organs such as the liver, lung, bone, or lymph node.

Metastasis involves complex multi-step cellular remodeling to enable tumor cells to leave their primary site and translocate to a secondary site [19]. It is estimated that human PCa cells exit from their primary site (local invasion, involving downregulation of cell–cell and cell–matrix adhesion which regulates extracellular matrix (ECM)), migrate into the surrounding tissue, and intravasate into the vasculature, which involves break down of the ECM using degradative enzymes to breach endothelial barriers [19]. In the vasculature, the cells survive as ‘circulating tumor cells’, and eventually extravasate into distal organs [20] (Figure 3).

To achieve the latter, the cell must arrest at a secondary endothelial site, bind to the endothelium, extravasate and transmigrate through the endothelial layer to reach the interstitium, where it coalesces with other metastasized cells to form a micrometastasis. Finally, the micrometastatic tumor cells colonize the distant site (Figure 3). In PCa patients, large numbers of cancer cells are released in circulation daily; however, <0.1% of them metastasize to distal sites [21]. The ultimate ‘seeding’ of metastatic cells requires conditions amenable for growth of the tumor [22].

The AR has a prominent role in the metastatic spread of PCa cells. One pathway that regulates AR-induced metastasis is the Rho small GTPase-regulated signaling pathway. Members of the Rho GTPase family are involved in AR signaling pathways that promote prostate cancer cell migration, invasion, and metastasis [23]. Rho GTPases control the actin cytoskeleton and can directly interact with and activate the AR, promoting androgen-independent growth of PCa cells [24]. This interaction will be investigated in this review.

### 2.3. PCa Treatment and Progression to CRPC

Despite substantial initial response to ADT, many patients cease to respond to treatment and develop castration-resistant PCa (CRPC) [1,6,15]. Gene expression profiles show that most (but not all) CRPC tumors maintain AR-dependency (despite DHT independence) by mutations in the AR-LBD which permit promiscuous ligand binding [25,26], and may even synthesize androgens through alternative pathways to reactivate LBD-expressing AR [27]. A third way of promoting CRPC is by the development of AR splice variants lacking the LBD [28] (Figure 1). AR-dependent CRPC is therefore initially treated with the ARSIs mentioned earlier—abiraterone acetate and the AR inhibitors (enzalutamide, apalutamide, darolutamide). Additional treatments in CRPC include chemotherapy (docetaxel, cabazitaxel), radiation (Radium-223, Lutetium Lu 177 vipivotide tetraxetan), immunotherapy (Sipuleucel T), or poly-ADP ribose polymerase (PARP) inhibitors [1,15].

Many patients with CRPC rapidly develop metastases—56% men with CRPC were diagnosed with metastatic disease (mCRPC) within 36 months of CRPC diagnosis [29]. There could be two major causes for this development. First, it is likely that micrometastases already exist in situ or as circulating tumor cells in the vasculature and, in an androgen-independent environment, these circulating groups of cells coalesce and grow into clinically detectable macrometastases [30]. Alternately, migratory properties of the malignant cells may be enhanced as a direct consequence of the treatment they undergo [31].

### 2.4. Membrane Androgen Receptors

Membrane receptors that bind androgens (mARs) are cell-surface receptors that mediate rapid, “nongenomic” effects of androgens, distinguishing them from the slower, genomic effects of AR [32]. Instead of altering gene transcription, mARs quickly modulate intracellular signaling cascades. Many of the non-genomic activities of androgens are likely through these receptors, which include T and DHT-activated G-protein coupled receptors (GPCR) involved in the regulation of skeletal muscle strength [33], anti-gonadotropic effects, sexual receptivity, and behavior [34]. A number of mARs have been identified—including GPR133 (ADGRD1), an adhesion GPCR (aGPCR) encoded by HCaR1, which binds to 5α-dihydrotestosterone (5α-DHT); ZIP9 (SLC39A9), a zinc transporter protein that influences apoptosis in PCa; GPRC6A, a GPCR expressed in PCa that is activated by L-α-amino acids and is modulated by calcium; OXER1, an oxoeicosanoid receptor expressed in PCa that binds testosterone and inhibits cell proliferation and migration; and TRPM8, an ion channel/transporter that is regulated by androgens [32]. Many of these androgen-binding GPCRs are expressed in so-called “AR-null” cells—for example, SLC39A9 and HCaR1 are expressed in both DU-145 and PC-3, while DU-145 cells also express small amounts of OXER1 (as reported in *Human Protein Atlas* [35]). AR-positive LNCaP cells express ADGRD1 and SLC39A9. Note that AR inhibitors do not affect the functionality of these GPCRs.

In addition to these GPCRs, the classic AR can also be palmitoylated and transported to the plasma membrane [36]. Specific isoforms of the classic AR, like AR45, that lacks the entire region encoded by exon 1 of the AR gene and is composed of the AR DNA-binding domain, hinge region, and ligand-binding domain, localize to the plasma membrane and play a role in calcium signaling (Figure 1) [37]. mAR activation can cause a rapid increase in intracellular calcium, initiate second messenger cascades, and activate protein kinases such as Src, Akt, and ERK [38]. Activating mARs induces apoptosis and inhibits migration and growth in PCa. Additionally, mARs cooperate with classic nuclear ARs by activating heat shock protein 27 (HSP27), which helps shuttle more AR into the nucleus and plays a role in reproductive physiology, endothelial vasodilation, and skeletal muscle strength [39]. mARs are implicated in the progression of CRPC and can trigger various signaling pathways, which influences the organization of the actin cytoskeleton [38]. The mAR’s distinct role and activation pathways make it a potential target for developing new drugs to treat PCa, especially CRPC.

**Figure 4:**
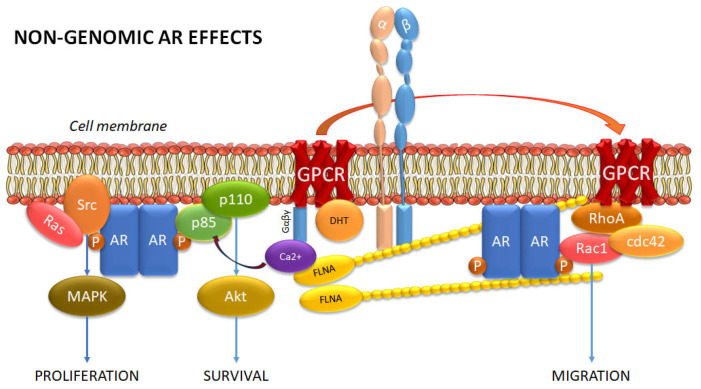
Non-genomic effects of androgens are rapid cellular responses, occurring within minutes, that do not involve direct gene transcription. These effects of androgens (both testicular and adrenal) occur mostly at the plasma and other membranes and are mediated by not only classical AR protein complexes but also by GPCRs that bind certain androgens, especially adrenal androgens. This results in activation of intracellular signaling pathways, such as Src, MAPK, and PI3K, leading to changes in protein phosphorylation, intracellular calcium levels, and cell proliferation. Ca^2+^ mobilization also leads to activation of Ca^2+^-activated scaffolding proteins such as Filamins that in turn activate integrins and Rho small GTPases that regulate proliferation, survival, and metastasis.

Studies show that in the absence of androgen binding, the AR may continue to affect downstream targeting through various means. First, the AR may undergo conformational changes that allow it to bind to non-specific ligands [40]. Second, AR transcriptional activity may also be regulated by various co-regulators, such as cyclin D1b and multiple HSPs, in a ligand-independent manner [41]. But a third way also exists—that includes non-genomic signaling by mAR at the plasma membrane [42] (Figure 4). Phosphorylation of the AR is required for multiple genomic functions, including for AR dimerization at the ARE [43]. However, mAR phosphorylation at the plasma membrane has been attributed to non-genomic regulation of sarcoma viral oncogene non-receptor tyrosine kinase (Src) and phosphatidylinositol 3-kinase (PI3K), leading to activation of mitogen-activated protein kinase (MAPK) and Akt (also known as protein kinase B or PKB), respectively, which in turn is known to mediate cellular functions such as proliferation and survival [43]. The AR also binds to actin binding proteins such as Filamin A, which is known to regulate actin binding to small GTPases of the Rho family that mediate cell migration and invasion [44]. (A cleaved form of Filamin A is also thought to inhibit AR transcriptional activity in the nucleus [45]). It is likely that these pathways are greatly upregulated when AR ligand binding is inhibited by ADT or the AR inhibitors. Indeed, studies indicate greater AR accumulation in the cytoplasm upon treatment with AR inhibitors such as enzalutamide which may thereby inadvertently stimulate greater migration and invasion upon development of resistance to ADT and the ARSIs leading to post-treatment mCRPC by activation of mARs [46].

## 3. Rho Family GTPases in PCa Progression

### 3.1. Structure and Classification of Rho Small GTPases and Regulators

**Figure 5:**
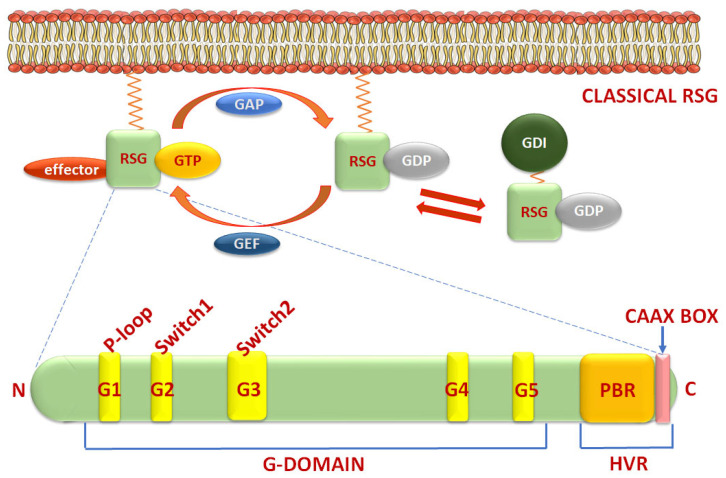
General RhoGTPase structure and mechanism of its cycling. Classical RSG are regulated by constant cycling between an active GTP-bound to an inactive GDP-bound state. In this effort they are aided by GEFs, GAPs, and GDIs. In contrast, atypical RSGs are regulated mostly by post-translational mechanisms, which are also seen in classical RSGs. Post-translational modification and expression patterns of Rho proteins as determined by -CAAX prenylation signal sequence at the carboxy terminus of RSG proteins; potential palmitoylation which may occur prior to the -CAAX sequence (not pictured). The CAAX sequence signals for geranylgeranyl, farnesyl, or truncated isoprenoid modification of the cysteine residue per specificity of each RSG. Prenylation of the carboxy terminus is made possible through proteolytic cleavage of AAX residues. The likely carboxylmethylation (OMe) of the now-terminal cysteine residue is not pictured. HVR—hypervariable region; PBR—polybasic region.

Small GTPases bind and hydrolyze guanosine triphosphate (GTP) to guanosine diphosphate (GDP), resulting in the phosphorylation of a downstream target. Small GTPases are molecular switches that go from a GTP-bound active to a GDP-bound inactive form and back (Figure 5) [47]. RSGs are essential in maintaining a tightly packed epithelial cell shape and polarity in cell migration via cytoskeleton rearrangement [48,49]. They help regulate distinct apical and basolateral domains with asymmetrical protein distribution to establish the adherens and tight junctions that determine cell shape, link adjacent epithelial cells, and form barriers between them to prevent diffusion of proteins [48]. RSGs work in unison within the cell to direct lamellipodial protrusions, actin–myosin contractions, and actin polymerization [49].

There are five similarity and function-based classes in the Ras superfamily of small GTPases—Ras, Rho, Rab, Ran, and Arf [50]. The small GTPases are characterized by the presence of G boxes in the G domain at the N-terminal and a hypervariable region (HVR) at the C-terminal which includes a polybasic region (PBR) and a C-terminal motif that determines its prenylation [51]. The G domain is made of five α-helices and a six-stranded β-sheet; it has been divided into five conserved sequence motifs G1–G5. The G1 motif (P-loop) recognizes the β-phosphate of the GTP and a Mg^2+^-ion on target nucleotides (Figure 5). The G2 motif (Switch1) binds the GTP γ-phosphate and a second Mg^2+^-ion. The G3 motif (Switch2) mediates GTP hydrolysis. The G4 and G5 motifs ensure binding to the guanine base vs. other nucleotides. The HVR is a flexible region at the C-terminal domain [51]. Within this region, the PBR, comprising multiple lysines or arginines, regulates protein interactions and membrane association and can also be a site of nuclear localization signal (NLS) sequences if the small GTPase is nucleus-bound. At the very C-terminal is the CAAX box, the site of post-translational modifications [52].

**Figure 6:**
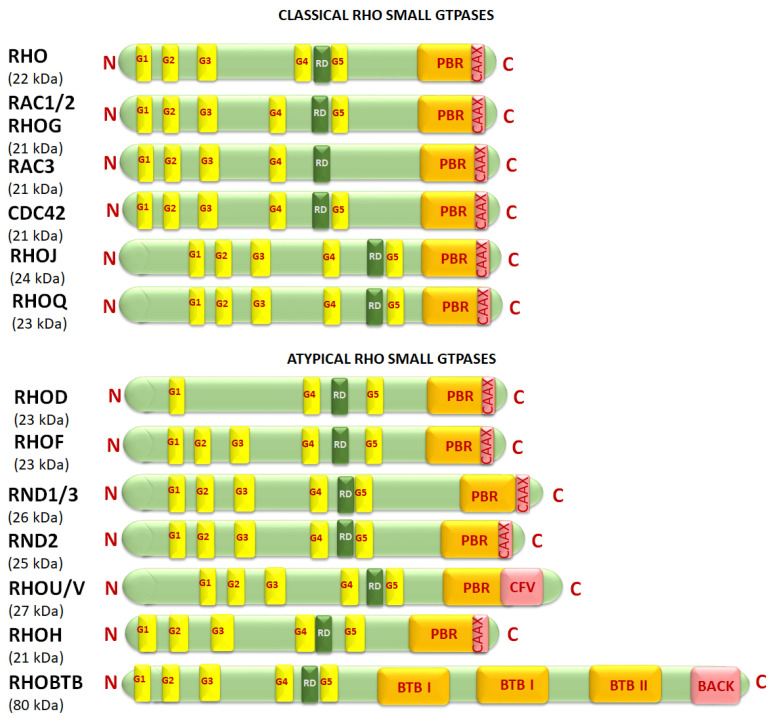
Differences in structure of members of the Rho family of small gtpases. The G domain of each Rho small GTPase is differentially structured. However, some subfamilies are equipped with additional domains that substantially affect their function. For example, RhoBTBs contain three BTB domains not seen in other subfamilies. RD—Rho insert region. PBR—polybasic region, CFV—cysteine–phenylalanine–valine sequence instead of CAAX sequence for palmitoylation. BACK—BTB and C-terminal Kelch domain (cullin 3-dependent ubiquitin ligase complexes) to prevent lipidation.

A key distinguishing feature in Rho small GTPases not found in other small GTPases of the Ras superfamily is the insertion of an approximately 13-amino acid α-helix called the “Rho insert region” between the G4 and G5 conserved sequence motifs in Rho GTPases [53] (Figure 6). The Rho insert region (sometimes called a Rho domain) plays a crucial role in binding to and activating specific effector proteins such as NADPH oxidase, IQGAP, ROCK, and mDia, and is a primary determinant of Rho-specific signaling pathways [54]. The Rho insert region is also involved in guanine nucleotide exchange factors (GEF) binding and is different in the different isoforms [54]. In Rac1 and RhoH it is shorter (6–7 aa), whereas in RhoBTB it is longer (18 aa) [55]. Differences in the CAAX box leads to a difference in isoprenylation (Table 2). In addition to isoprenylation by farnesylation and geranylgeranylation, RSGs are also sometimes subjected to palmitoylation (e.g., RhoB, Cdc42, RhoJ, RhoQ, RhoU, RhoV) [52]. Palmytoylation, similar to isoprenylation, affects RSG localization, activity, and interaction with other proteins [52].

GTPase activity of RSGs is inherently low; hence regulatory proteins are required for switching of classical RSGs between active GTP-bound and inactive GDP-bound states. The interchange of GDP with GTP is facilitated by GEFs, which activate the signaling pathway downstream of RSGs [56]. The reverse interchange of GTP with GDP is facilitated by GTPase-activating proteins (GAPs), which are required to terminate the signal (Figure 5) [57]. Other regulators—the guanosine nucleotide dissociation inhibitors (GDI) and their inhibitors, the GDI displacement factors (GDF)—also regulate RSG function [58]. Isoprenylated small GTPases can be extracted by GDI from the membrane by binding to the prenylated inactive forms whereby GDI forms a complex with its target GTPase and confines it to the cytosol. The activity and stability of the GTP-bound RSG can be affected by binding to various effector proteins that can also induce downstream signaling.

### 3.2. Regulation of Metastasis by the Rho-GTPase Family in PCa

Here, we will discuss how each subfamily of the Rho family of small GTPases regulates PCa progression, remembering that not all members have been found to have specific roles in this context. Both classical and atypical RSGs will be discussed (Figure 6). Overall, we see that the classical RSGs (Rho, Rac, Cdc42) have been shown to have significant oncogenic and metastatic properties in PCa, whereas atypical RSGs (Rif, Rnd, RhoBTB) have not been similarly examined. The exceptions are RhoH and RhoU, which promote metastasis and/or oncogenesis in collaboration with Rac1 or Cdc42.

#### 3.2.1. RhoA/B/C Subfamily

The major function of the Rho subfamily in PCa is to regulate motility, migration, and invasion by modulating the actin cytoskeleton. RhoA, RhoB, and RhoC share ~85% sequence homology but differ in sequence near the C-terminus, which alter their expression profile, regulation, localization, and functional activity [59,60]. Isoprenoid modification of the cysteine residue in the C-terminus appears crucial in maintaining intrinsic GTPase activity. RhoA and RhoC, each derived from genes encoding seven exons, are prenylated only with a geranylgeranyl group [59,60] (Table 1). Rho B, on the other hand, is encoded by a gene consisting of only one exon [61]. RhoB can be geranylgeranylated, farnesylated, or palmitoylated. RhoA and RhoC primarily anchor themselves in the plasma membrane, whereas RhoB can also localize to the membranes of late endosomes and lysosomes [4,59,60]. RhoA regulates actin–myosin contraction via the focal adhesion complex and stress fiber assembly when activated by extracellular ligands like lysophosphatidic acid (LPA) [62]. RhoC mediates endothelial cell interaction, invasion, metastasis, and vascularization in PCa and, prior to intravasation, it regulates PCa cell insertion between, and attachment to, endothelial cells [63] (Table 2). Binding partners of the Rho small GTPases are shown in Table 3.

The effects of RhoA on the actin cytoskeleton are mediated by Rho-associated coiled coil containing protein kinase (ROCK) [64] (Table 3). GTP-bound RhoA binds to the C-terminal Rho-binding domain (RBD) on ROCK, releasing its auto-inhibitory fold and activating its kinase domain. Activated ROCK then phosphorylates various downstream targets, including myosin light chain kinase (MLCK) and other proteins, leading to changes in cell shape, contraction, and migration. Y-27632 dihydrochloride is a ROCK inhibitor that binds to the catalytic ATP-binding pocket of ROCK, and prevents the protein from phosphorylating its downstream targets, including MLCK [65]. The primary isoforms of ROCK–ROCK1 and ROCK2 share 65% sequence identity, but have distinct roles in cell motility [66,67]. While ROCK1 mainly phosphorylates MLCK, ROCK2 also phosphorylates cofilin via integrin β1-activated FAK signaling [68]. Both these pathways, however, promote directional migration. Y-27632 inhibits both ROCK1 and ROCK2 and targets the oncogene c-Myc [69]. The Rho–ROCK pathway also plays a role in angiogenesis, is important in metastatic spread, and is generally upregulated in PCa (Table 4).

In contrast to RhoA, RhoB has a more tumor-suppressive role in PCa. It regulates cell survival and influences cell morphology, adhesion, transformation, invasion, and metastasis [61,70,71]. In AR-null PC-3 cells, RhoB regulates β1 integrin at the plasma membrane in the development of Rac1-mediated lamellipodium and cell adhesion [61]. It co-localizes with E-cadherin to stabilize cell–cell adhesions of another AR-null cell line, DU145. RhoB reduction increases N-cadherin expression to promote cell migration. RhoB suppresses EMT, promotes chemosensitivity and prevents migration and invasion in PC-3 cells [72]. The RhoB inducer NSC126188 induces apoptosis in PC-3 cells by inhibiting Akt and its downstream target FOXO3 and the reactive oxygen species (ROS)-mediated c-Abl/p38 MAPK signaling [73]. RhoB also upregulates cell–cell adhesion and E-cadherin expression while decreasing migration in DU145 cells as well as in PC-3 cells [74].

However, in AR-positive tumors, it is likely that AR status plays a role in the differential effect of RhoB as a tumor suppressor vs. tumor promoter. One study indicates that in AR-positive PCa cells, those expressing AR variants (AR-V) observe higher RhoB levels that promotes PCa progression, whereas full-length AR represses it [75]. Therefore, RhoB is likely a tumor suppressor that turns into an oncogene in different cellular contexts.

Other studies indicate that RhoA and RhoB mediates non-genomic effects of the AR, likely via mAR. In HSPC LNCaP cells, activation of RhoA/B and ROCK is implicated in mAR-dependent actin polymerization and apoptosis (T had a similar effect in DU145 cells that lack classical AR but express mARs) [65]. RhoB also regulates Rac-mediated spreading and lamellipodium extension, but not membrane ruffling [61]. Depletion of RhoB results in cell rounding due to reduced surface β1 integrin [61]. It is likely that AR-V expression is responsible for the differential effect of RhoB on some AR-positive tumors. Analysis of TCGA data shows that unlike RhoA and RhoC, RhoB is significantly decreased in PCa (Table 4). One study indicates that AR-positive PCa cells expressing AR-V express higher RhoB that promotes PCa progression, whereas full-length AR represses it [75]. Therefore, it is likely that RhoB is a tumor suppressor that turns into an oncogene in different cellular contexts.

In contrast, RhoC is unambiguously an oncogene with a twist—it has little effect on tumor growth or proliferation but instead plays an oversized role in metastasis in PCa by sequential activation of Pyk2, FAK, MAPK, and Akt, followed by the upregulation of MMP2 and MMP9 [76]. Other studies show that it affects invasion but not motility in PC-3 cells [77]. RhoC increases PCa relapse compared to localized tumors [78], while TCGA data show that it decreases disease-free survival (Table 4). RhoC is such an important factor in PCa metastasis that a vaccine-targeting RhoC (RV001) has been devised in patients with localized PCa to prevent distant metastases [79]. RhoC also regulates the interaction of PCa cells with vascular endothelial cells (ECs), a crucial step in the metastatic process that promotes angiogenesis, via activation of ROCK1 and ROCK2 [63]. Drugs currently available against various members of the Rho family are shown in Table 5.

#### 3.2.2. Rac Subfamily

The Ras-related C3 botulinum toxin substrate (Rac) subfamily (Rac1, Rac2, Rac3, RhoG) is also a master regulator of cell motility and invasiveness and is highly overexpressed in CRPC. Rac1 is implicated in cell cycle progression, cell migration, gene expression, and increased apoptosis [80]. In most cancers, these properties of Rac1 are attributed to gain-of-function mutations in the Rac1 gene; however, in PCa, Rac1, like most other Rho family members, is rarely mutated, hence tumorigenesis may be attributed to associated regulatory proteins. Rac1 promotes motility by inducing lamellipodia formation crucial for cell migration and movement, allowing cells to explore their environment and move towards specific stimuli [81]. It is required for PC-3 cell diapedesis across a bone marrow endothelial cell layer [82]. Rac1 has also been shown to play a role in cell survival by regulation of Akt-mediated phosphorylation of BCL2-associated agonist of cell death (BAD) [83]. Platelet-derived growth factor (PDGF), EGF, integrin-mediated extracellular matrix adhesion, or insulin may activate Rac1 to form leading edge lamellipodia, resulting in a motile cell surface, and/or promoting development of nascent focal complexes through PI3K activation [84,85,86]. Additionally, Rac1 and RhoA regulate one another as they impact alternate cell processes [87]. Inhibition of Rac1 also prevents enzalutamide resistance in CRPC [88]. A number of Rac1 inhibitors have been synthesized (NSC 23766, 1A-116, Z62954982, EHT 1864 2HCl, EHop-016), but have yet to be successful in clinical studies [89] (Table 5). Of these, NSC 23,766 showed efficacy in PCa [90]. Rac1 binds and activates effectors like p21-activated kinase 1 (PAK1), a serine/threonine kinase of the 6-member PAK family that plays a crucial role in regulating the actin cytoskeleton [81].

Rac2 is primarily expressed in hematopoietic cells, including neutrophils [91]; however, some studies indicated that it is downregulated in localized PCa compared to BPH [92], while others found the opposite [93]. In contrast, Rac3 small GTPase [not to be confused with receptor-associated co-activator 3 (RAC3)] is upregulated in PCa [82] (Table 4). RhoG also has a role in PCa cell adhesion and invasion in both PC-3 and C4-2 cells [82].

#### 3.2.3. Cdc42 Subfamily

Cdc42 is also overexpressed in PCa and is implicated in cell cycle progression, cytoskeletal organization, migration, and increased apoptosis [90]. Cdc42 shares many effectors with Rac1 such as PAK1 and is implicated in the regulation of telomerase activity [90,94]. Significantly, Cdc42, along with PI3K, is involved in tyrosine kinase inhibitor-induction of entosis, a homogeneous cell-in-cell phenomenon and a non-apoptotic cell death process that involves the upregulation of E-cadherin and the downregulation of ROCK [95]. Cdc42 inhibition in both androgen-dependent and androgen-independent cell lines shows reduced tumorigenesis through decreased cell filipodia formation, proliferation, migration, and impediment of cell cycle progression [96,97].

Cdc42 is important in pseudopodia formation, which is instrumental in cell invasion and migration and is also a prominent mediator of angiogenesis, thus contributing to PCa progression [98,99]. It activates β1-integrin via extracellular signal-regulated kinase (ERK)/FAK signaling at the protein level and serum response factor (SRF)-induced transcriptional activity [100]. Cdc42 subsequently induced PAK1 phosphorylation and upregulation of Matrix Metalloproteinase-9 (MMP-9), which regulates tissue remodeling, inflammation, and wound healing [101]. MMP-9 is a protease that degrades components of the ECM, like type IV collagen, laminin, and fibronectin, thereby promoting cytoskeletal restructuring [102]. Cdc42 levels were higher in the serum of patients with mCRPC compared to healthy controls and correlated with lymph node and visceral metastasis [103]. Significantly, treatment with ABI decreased Cdc42 levels after 2 months [103]. A reduction in Cdc42/GSK-3β/Snail and induction of E-cadherin levels accompany a change in cell morphology to a more epithelial-like shape in DU145 cells, indicating a role for this protein in EMT [104]. A number of Cdc42 inhibitors have been developed (MBQ-167, ZCL-278, MLS-573151, CID44216842, ML141, ARN25062) (Table 5)– of these, only ML141 and ZCL278 have been tested in prostate models [105,106]. The other two members of this subfamily—RhoJ and RhoQ—have not been reported to have any role in these processes in PCa; however, TCGA data indicate that RhoJ is significantly decreased in PCa (Table 4).

#### 3.2.4. Rif Subfamily

The two known members of this family are RhoD and RhoF, which impact cell morphology, but associations of these RSGs with PCa are not fully understood as yet. RhoD and RhoF were initially thought to be classical RSGs, but later studies confirmed their atypical status. RhoD impacts intracellular compartmental vesicle trafficking and endosomal motility while influencing actin cytoskeleton dynamics, including loss of stress fibers, focal adhesion disassembly, and actin-based protrusions at the plasma membrane [107]. Semaphorins, proteins involved in neuronal development, bind to plexins and neuropilins, which have been implicated in PCa metastasis and progression [108]. Mutations in plexins promote RhoD binding which impaired trafficking of plexinB1 to the membrane [109]. A mutation in RhoD, RhoD (G26V), antagonized RhoA [110]. These studies showed that RhoD is an anti-migratory factor. TCGA data indicate that high RhoF expression poses a significant hazard to disease-free survival in PCa (Table 4).

#### 3.2.5. Rnd Subfamily

The atypical Rnd subfamily (Rnd1, Rnd2, Rnd3) plays a role in PCa by mediating the effects of the semaphorin ligands, namely the plexins [111]. PlexinB1 (encoded by *PLXNB1*) is implicated in both migration and invasion of PCa cells [112]. Plexins express an RBD that acts as a GAP for Rnds. Mutations in plexins are found to promote their binding to Rnd1 which impairs trafficking of plexinB1 to the membrane [111]. Mutations in the RBD of plexinB1 results in a complete loss of Rnd1 binding, promoting oncogenesis.

While Rnd2 is yet to have an identified role in PCa, Rnd3 (also called RhoE) is expressed in the prostate and is upregulated by NF-κB [113]. Rnd3 is expressed at higher levels in benign prostate and in stromal cells, compared to PCa [114], and is higher in HSPC compared to CRPC [115]. Moreover, Rnd3 mRNA and protein expression were significantly reduced in PCa compared to benign tissue [116] (Table 4). Rnd3 overexpression in LNCaP cells induces G2/M arrest and increases apoptotic cell death [116,117]. It is also considered to be a cell adhesion gene that prevents metastasis. Thus, this subfamily of atypical RSGs exhibit a tumor-suppressive function in HSPC [115].

#### 3.2.6. Wrch Subfamily

The Wrch subfamily consists of two atypical family members, RhoU, also known as Wnt-regulated Cdc42 homolog-1 (Wrch1), and RhoV, also known as Cdc42 homologous protein (CHP, also called Wrch2). RhoU has significant oncogenic properties and its silencing in PC-3 and VCaP cells by siRNA resulted in marked growth arrest and cytotoxicity in 3D organotypic cell culture conditions but not in 2D culture [118]. In PC-3 organoids, it also resulted in reduced tumor cell invasion. RhoU also independently predicted PSA recurrence better than a model that included Gleason grade and tumor stage [119] (Table 4). Because of these properties, RhoU has been repeatedly used as a biomarker to predict the presence of PCa tissue in histologically benign prostate tissue [119,120,121].

RhoU is one of the rare atypical RSGs that is only palmitoylated and not isoprenylated (Table 2), and its palmytoylation is highly regulated by fatty acid synthase (FASN) [122,123], which is itself commonly overexpressed in PCa and is associated with tumor progression [124]. FASN regulation of RhoU palmitoylation influences cell adhesion [122,123]. Cdc42 palmitoylation is dependent on the palmitoylation status of RhoU. C-terminal palmitoylation of RhoU is required for its homodimerization through its C-terminal extension which affects RhoU-induced PAK1 activation, which in turn induces cell morphological changes [124]. RhoV is oncogenic in lung cancer and triple-negative breast cancer [125]; however, in PCa, higher RhoV expression is associated with a favorable prognosis [126]. RhoV is downregulated in localized PCa compared to non-tumor tissue. It is expressed in DU145 cells when co-cultured with normal prostate fibroblasts [126].

#### 3.2.7. RhoH Subfamily

RhoH is currently the sole member of this family of atypical RSGs and lacks GTPase activity as it is unable to hydrolyze GTP, relying instead on protein localization and changes in protein levels for regulation [127]. It is also oncogenic. RhoH expression, normally restricted to hematopoietic cells, is frequently genetically rearranged in multiple epithelial cancer cell lines [127]. Increased RhoH expression in PCa correlates with earlier relapses following prostatectomy (Table 4), whereas RhoH depletion reduces cell migration [127]. RhoH interacts with Rac1 and PAK1 to regulate lamellipodium extension. Rac1-induced lamellipodium formation at the leading edge requires RhoH activation [127]. In the absence of RhoH, Rac1 induces lamellipodia all around the cells, which severely affects the ability of the cells to undergo directional movement during migration and invasion. Therefore, RhoH is essential for Rac1-induced metastasis.

#### 3.2.8. RhoBTB Subfamily

The RSG RhoBTB1 contains two BTB domains [also known as POZ (poxvirus and zinc finger) domain, crucial for protein–protein interactions] along with a Rho domain (Figure 6). RhoBTB1 mediates actin cytoskeletal rearrangement but does not directly impact cell shape or motility [128]. This protein has significant tumor-suppressive properties in PCa and is downregulated in PCa (Table 4). RhoBTB1 depletion leads to increases in invasion and elongation in PCa [128]. It interacts with and inhibits ROCK1 and ROCK2 to suppress cancer cell invasion [128]. RhoBTB1 is primarily localized to the cell’s Golgi apparatus and vesicular structures. Very little has been reported on RhoBTB2 in PCa.

### 3.3. Regulation of Classical Rho Small GTPases in PCa

Aberrant receptor tyrosine kinases (RTK) or GPCR signaling leads to GTPase activation of downstream effectors. Another cause of GTPase activation is upregulated/mutated regulator activity. As the intrinsic GTPase activity of classical RSGs is not efficient or rapid, regulatory molecules such as GEFs, GAPs, and GDI are utilized to accelerate GDP–GTP exchange and GTP hydrolysis [129]. RSGs respond to physical and mechanical cues and work together to regulate processes such as adhesion, movement, and cytoskeletal rearrangement. In contrast, atypical RSGs are almost always constitutively active, and are regulated by post-translational modifications such as phosphorylation or lipidation.

#### 3.3.1. Role of GEFs in Rho GTPase Activation

**Figure 7:**
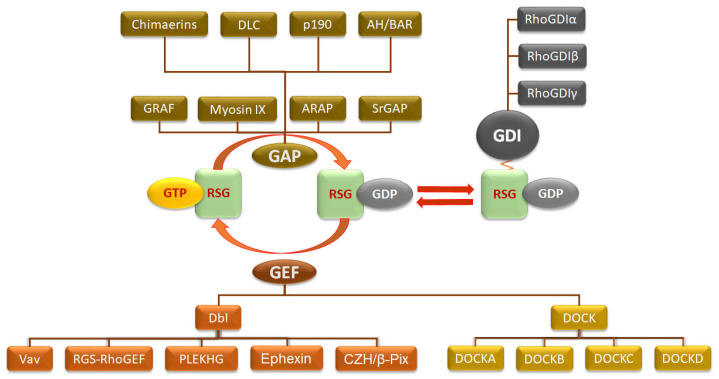
Classification of RhoGEFs, RhoGAPs, and RhoGDIs. RhoGEFs are classified into two main categories—the Dbl family and the DOCK family. The Dbl family contains a tandem Dbl homology (DH) catalytic followed by a pleckstrin homology (PH) regulatory domain topology, while the DOCK family contains two high sequence regions (DOCK-homology region) called the regulatory DHR-1 and catalytic DHR-2 domains. A couple of other RhoGEFs have been identified that do not follow under either of these families—namely, SWAP70 and SLAT, which contain a PH but no DH domain. RhoGAPs contain a core RhoGAP domain, but in addition, Chimaerins contain a phorbol ester-binding C1 domain, the DLC subfamily (DLC1, DLC2, and DLC3) features a Sterile Alpha Motif (SAM), and a StAR-related lipid-transfer (START) domain, the P190 family (P190-A and P190-B) contains a GTP-binding domain, two pseudo-GTPase domains, the AH/BAR family contains an N-terminal Armadillo-related/ANTH-like Homeodomain (AH) domain or a Bin-Amphiphysin-Rvs (BAR) domain. The Myosin IX subfamily (Myosin IXA and Myosin IXB) are unconventional myosins and the ARAP subfamily contains ArfGAP domains and multiple PH domains, while SrGAPs contain an F-BAR domain and an SH3 domain. There are three members of the RhoGDI family in humans: RhoGDI1 (RhoGDIα), RhoGDI2 (D4-GDI/RhoGDIβ), and RhoGDI3 (RhoGDIγ).

Activation by GEFs is widely considered the most common and fundamental mechanism for the signal-mediated activation of small GTPases [129]. Currently, there are ~83 known GEFs for RSGs, of a total of about 150 [130]. The GEFs specific for RSGs (RhoGEFs) are divided into two main families (Figure 7)—the Dbl family (n = 70) that contains a Dbl homology (DH) catalytic and a pleckstrin homology (PH) regulatory domain, and the DOCK family (n = 11) that contains two high sequence regions (DOCK-homology region, DHR), the regulatory DHR-1 and catalytic DHR-2 domains. A couple of other RhoGEFs have been identified that do not fall under either of these families—namely, SWAP70 and SLAT, which contain a PH but no DH domain [131]. Subfamilies within the Dbl RhoGEFs include the Vav family (Vav1, Vav2, Vav3); the RGS family (p115RhoGEF, LARG, PDZ) that link signals from G12/13 family G proteins to RSGs; the PH domain-containing RhoGEF family (PLEKHG1-7); the Ephexin family consisting of five members (Ephexin1–5) that link the RTK Eph to the cytoskeleton; and the CZH family, that includes β-Pix [132]. The DOCK family primarily activate Rac and Cdc42 but not Rho [133]. DOCK proteins fall into four subgroups (DOCK-A, -B, -C, and -D) with varying GTPase specificities and domain architectures [134].

RhoGEFs activate classical RSGs of the Rho, Rac, and Cdc42 subfamilies [135]. In Dbl RhoGEFs, the DH domain is composed of an extended bundle of α-helices composing three conserved regions—CR1, CR2, and CR3, forming key contact points with the RSG [136]. It is the catalytic site that drives nucleotide exchange, binding to the inactive, GDP-bound form of the RSG and promoting the release of GDP. This allows a GTP to bind to the RSG, activating it. The DH domain contains the GEF switch-specific amino acid residues and structural features that interact with and activate particular RSG, ensuring specificity in the signaling pathway [137]. The regulatory PH domain is expressed C-terminal to the DH domain [138]. It binds to phospholipids in the plasma membrane, thereby targeting the RhoGEF to the inner membrane [139,140]. The PH domain also interacts with RSGs, thereby ensuring the binding of the RSG to the membrane [141]. The PH domain associates with phospholipids and folds over; this controls the activity of the DH domain [141]. There are several other domains found in individual RhoGEFs that vary depending on their individual function and the unique sequence of the protein.

Studies showed that the RhoGEF Vav2 is elevated in enzalutamide-resistant cells compared to enzalutamide-sensitive ones. Vav2 overexpression promotes PCa proliferation and metastasis by activating the PAK1/Akt pathway, and stabilizing AR/AR-V7 binding [142]. Another RhoGEF of the same family, Vav3, also correlated positively with PCa cell migration and invasion [143]. Vav3 is phosphorylated by the ephrinA1 receptor EphA2, which activates Rac1, increasing migration and invasion. Expression of Vav3 and EphA2 is increased in advanced PCa and correlates with lower time to progression, indicating a role in PCa metastasis [143]. The roles of other RhoGEFs in PCa are unknown.

#### 3.3.2. Role of GAPs in Rho GTPase Inactivation

RhoGAPs are negative regulators of RSGs that accelerate the hydrolysis of GTP to GDP, thereby switching the RSG from its active “on” state to its inactive “off” state. There are ~67 known RhoGAPs [130]. RhoGAPs bind to activated Rho GTPases and enhance the GTPase activity, leading to GTP hydrolysis and the inactivation of the GTPase. While some RhoGAPs act on multiple Rho GTPases, others are highly specific in their target RSGs [144]. All RhoGAPs share a conserved catalytic RhoGAP domain, approximately 150 amino acids long, which is the main domain responsible for accelerating GTP hydrolysis [145]. Additionally, RhoGAPs contain a lipid-binding domain such as a PH domain which targets the protein to specific membranes, protein-interacting domains such as the SRC homology 3 (SH3) domain which influences regulation, and a catalytic domain with different enzymatic activities that can modulate RhoGAP function [51].

RhoGAPs can be classified based on the functional domains they express beyond the core RhoGAP domain (Figure 7) [146]. Chimaerins contain a phorbol ester-binding C1 domain. α- and β-chimaerins have GAP activity specifically for Rac, but not Rho or Cdc42. The Deleted in Liver Cancer (DLC) subfamily (DLC1, DLC2, and DLC3) are tumor suppressors and feature a Sterile Alpha Motif (SAM), the RhoGAP domain, and a StAR-related lipid-transfer (START) domain. The P190 family (P190-A and P190-B) are large multidomain proteins that include a GTP-binding domain, two pseudo-GTPase domains, and the RhoGAP domain. Their activity is regulated by phosphorylation. The AH/BAR family is characterized by the presence of an N-terminal armadillo-related/ANTH-like homeodomain (AH) domain or a Bin-Amphiphysin-Rvs (BAR) domain. A subgroup of AH/BAR is the GRAF subfamily (GRAF1, GRAF2, and GRAF3) that contain a BAR domain, a PH domain, and an SH3 domain and associate with FAK. The Myosin IX subfamily (Myosin IXA and Myosin IXB) are unconventional myosins that have a RhoGAP domain, while the ankyrin repeat and PH domain (ARAP) subfamily contain both RhoGAP and ArfGAP domains, linking the regulation of Rho and Arf GTPases. They also contain multiple PH domains. Finally, the Slit-Robo GAPs (SrGAPs) are critical for neuronal migration and contain an F-BAR domain, a RhoGAP domain, and an SH3 domain. However, other RhoGAPs exist that are not part of any specific subfamily.

One such RhoGAP is ARHGAP21, which acts specifically on RhoA, RhoC, and Cdc42 [147]. ARHGAP21 is located in the nucleus and cytoplasm of LNCaP and PC-3 cells; but knockdown of ARHGAP21 decreases proliferation and increases migration only in PC-3, where it demonstrates GAP activity for RhoA and RhoC and induces changes in cell morphology [147]. Other than a RhoGAP domain, it also contains a PH domain and an Arf-binding domain (ABD) [148]. In an animal model of PCa, it regulates tumor growth [149]. ARHGAP29 (also called PARG1) is a member of the AH/BAR family that contains a C1 domain, an F-BAR domain and a coiled coil region in addition to the RhoGAP; it is also associated with metastasis in PCa [150]. ARHGAP29 promotes cell proliferation and invasion and predicts PSA recurrence [150]. Another member of the AH/BAR family is SH3BP1, which drives cell motility in PCa [151], while ARHGAP5, encoded by p190 B, is also expressed in PCa and regulates cell growth [152]. ARHGAP5 contains a PBR that binds to acidic phospholipids and a divergent middle domain. The BAR domain is a lipid-binding module that senses and molds membrane curvature to coordinate membrane and cytoskeletal dynamics, thereby influencing the localization and activation of the RhoGAP domain and linking membrane trafficking with RSG signaling [153]. Therefore, it is likely that these proteins promote migration.

Other BAR-containing RhoGAPs, such as ARHGAP10 (GRAF2), are tumor-suppressive in nature. Increased expression of ARHGAP10 in PCa is associated with increased disease-free survival [154,155]. ARHGAP10 downregulates Cdc42 in PCa [154]. ARHGAP10 is downregulated in PCa and inversely correlates with Wnt signaling [155]. StarD13 (also known as DLC2) act on RhoA and Cdc42 [156]. DLC2/StarD13 is downregulated in PCa, and suppresses cell proliferation and cell adhesion, as well as cell migration, invasion, and matrix degradation through its regulation of Cdc42 [156]. DLC1 is mutated frequently in PCa [157] and affects cell adhesion [158]. DLC1 transduction negatively regulates NF-κB activity via IκBα phosphorylation [159]. Its interaction with α-catenin stabilizes adherens junctions, a cell–cell adhesion complex associated with the actin cytoskeleton [160]. It also increases E-cadherin expression by suppressing RhoA and RhoC [161]. This results in inhibition of angiogenesis in PCa tumors [162]. DLC1 contains an N-terminal SAM domain, a central RhoGAP domain, and a C-terminal Steroidogenic Acute Regulatory Protein (START) domain. It also contains an SR region with a focal adhesion targeting (FAT) domain and a serine-rich region, which likely contributes to its role as a tumor suppressor. Thus, RhoGAPs can have both tumor promoting and tumor-suppressive properties in a context-dependent manner.

#### 3.3.3. Rho GDP-Dissociation Inhibitor—RhoGDI in PCa

RhoGDI regulates the activity of Rho family GTPases by inhibiting nucleotide exchange and membrane association, effectively keeping them in an inactive GDP-bound state in the cytosol and preventing them from binding to GTP in the plasma membrane [163,164]. Thus, RhoGDI plays a crucial role in regulating the overall activity of RSGs. There are three members of the RhoGDI family in humans (Figure 7): RhoGDI1 (RhoGDIα), RhoGDI2 (D4-GDI/RhoGDIβ), and RhoGDI3 (RhoGDIγ) [163]. Each RhoGDI exhibits distinct patterns of tissue expression and binding specificities.

RhoGDIγ has a unique N-terminal extension that targets it to the Golgi complex and other membranes [165]. RhoGDIγ interacts mostly with the classical RSGs RhoB and RhoG by extracting them from the plasma membrane by binding to its prenyl moiety [164]. RhoGDIγ contains a hydrophobic pocket that accommodates this prenyl group, effectively pulling RhoG off the membrane [166,167]. Once extracted, RhoGDIγ sequesters RhoG in an inactive, GDP-bound state by forming a stable complex in the cytosol [165]. This binding prevents RhoG from interacting with RhoGEFs, which would normally activate it by replacing its GDP with GTP. RhoGDIγ then translocates inactive RhoG from the plasma membrane to the Golgi to sequester it in a non-functional state, protecting it from degradation and preventing its inappropriate activation.

Overexpression of RhoGDIβ, a gene associated with TMPRSS2:ERG fusion-positive PCa, elicits a spindle-shaped morphology, faster cell migration and increases cell proliferation, phenotypic changes suggestive of cancer progression [168]. In contrast, RhoGDIα was identified as a metastasis-inhibitory protein in PCa patients [169]. Expression of RhoGDIα is downregulated in more aggressive PCa cells compared to LNCaP [170]. Overexpression of RhoGDIα inhibits the growth of PCa cells and causes CRPC reversal to an androgen-sensitive state, while downregulation of RhoGDIα enhances growth of androgen-sensitive LNCaP cells in androgen-deprived conditions. In addition, RhoGDIα suppresses the tumorigenic ability of prostate tumor xenografts in vivo [170]. Taken together, it is likely that RhoGDIα associates with RSGs involved in cell growth and survival while RhoGDIβ associates with those involved in metastasis.

### 3.4. Regulation of Atypical Rho Small GTPases in PCa

Unlike classical RSGs, atypical RSGs are not regulated by the GEF/GAP/GDI pathway. They are regulated through alternative mechanisms, including post-translational modifications, transcriptional regulation, and intrinsic GTPase activity [171]. Some atypical RSGs have N-terminal extensions or other unique features that further influence their regulation and activity [172]. Rnd, RhoH, and RhoBTB are activated by phosphorylation, ubiquitination, and SUMOylation [173]. These proteins are GTPase-deficient and have a stalled GTPase activity.

In contrast, Wrch subfamily members are fast-cycling RSGs that freely cycle between GTP-bound and GDP-bound conformations without the involvement of RhoGEFs, RhoGAPs, or RhoGDIs [172]. The intrinsic GDP/GTP exchange activity in Wrch RSGs is about 10-fold higher compared to classical RSGs. As a result, the fast-cycling RSGs reside predominantly in an active conformation. The Rif members (RhoD and RhoF) are also faster cycling compared to classical RSGs [107]. These fast-cycling GTPases reactivate by rapidly releasing bound GDP through the actin nucleation factor Arp2/3 [174]. When first discovered, they were thought to be classical, since they have GDP/GTP exchange activity [175], but were reclassified as atypical when it was discovered that this activity is intrinsic, rendering the proteins constitutively active. Importantly, RhoD and RhoF can still hydrolyze GTP and an intact GTPase activity was required for efficient fusion of RhoD-positive vesicles [175]. RhoD has a unique N-terminal extension of 14 amino acid residues, which is not present in the classical RSGs [175].

### 3.5. Regulation of RSG Membrane Binding by Lipidation

Most of the RSGs, both classical and atypical, with the exception of RhoBTBs, are membrane-localized by a post-translational mechanism involving the covalent linking of an isoprenoid lipid to a cysteine residue at the C-terminal of the protein, which can then insert itself into the non-polar membrane [52]. There are two predominant groups of isoprenoid lipids that can attach themselves to RSGs—(i) a farnesyl group, consisting of 3 isoprene repeats with a total of 15 carbon atoms, or (ii) a geranylgeranyl group of 4 isoprenes and a total of 20 carbon atoms [176]. Both groups typically attach via a multi-step post-translational process involving thioester linkage with the C-terminal cysteine residue. There is also a non-isoprenoid lipid that is attached post-translationally to some RSGs, (iii) a 16-carbon palmitic acid, the most prevalent saturated fatty acid in humans [177].

The CAAX motif is a C-terminal tetrapeptide sequence with a cysteine (C), two aliphatic amino acids (A_1_, A_2_), and one of several specific amino acids in the terminal position (X). The CAAX motif determines substrate specificity for a farnesyl or geranylgeranyl transferase [52,178]. Eukaryotic proteins with a CAAX motif undergo a three step post-translational modification. First, the cysteine group is isoprenylated by either the C15 farnesyl transferase (FTase) or the C20 geranylgeranyl transferase I (GGTase I) (the X determines which transferase works—e.g., Leu and Phe favors geranylgeranylation). This is followed by cleavage of AAX by the proteases Rce1p and Ste24p [52]. Finally, the isoprenylated cysteine is carboxylmethylated by an isoprenylcysteine carboxyl methyltransferase (ICMT). These steps are necessary for the activity, subcellular localization, and stability of the modified GTPase.

The Wrch group (RhoU and RhoV), instead of a CAAX box, carry a DHHC motif (Asp-His-His-Cys), a key motif required for palmitoylation [179]. Palmitoylation of RSGs involves the reversible modification of cysteine residues by the saturated fatty acid palmitate, catalyzed by DHHC family palmitoyltransferases at cysteine residues near the CAAX box [52]. Palmitic acid is attached to a cysteine residue on the protein via a thioester linkage, catalyzed by a family of palmitoyltransferases (PAT), which are classified as acyltransferases. The palmitoylation modification is reversible, which is important for dynamic regulation of RSG function and localization [180]. This process is called S-palmitoylation and is carried out at the C-terminal of the RSG. RSGs do not undergo N-palmitoylation, which is undergone at the N-terminal. Some RSGs, such as Cdc42 and RhoB, can undergo both isoprenylation as well as palmitoylation [181]. Farnesylation is usually the first step, resulting in membrane binding. Palmitoylation then further enhances this membrane association. The more C-terminal cysteine residue within the CAAX motif is typically farnesylated, while the adjacent cysteine residue is palmitoylated.

### 3.6. Effects of Genetic Variants on Rho Small GTPase Function in Prostate Cancer

Unlike in other cancers, RSGs are rarely mutated in PCa, the majority of the alterations being caused by changes in transcription, or functional changes caused by their regulators. A 2017 study found that in RhoA, rs2410 mutant allele and rs2269736 wild allele were risk factors for PCa [182]. Specific mutations in RhoB are not reported but it is usually downregulated in PCa (Table 4) and its loss is associated with enhanced EMT [72]. Mutations in RhoC are also not reported widely, but its overexpression in PCa (Table 4) is associated with an increase in metastasis by activating a cascade of proteins, including Pyk2, FAK, MAPK, and Akt [76].

Like Rho RSGs, Rac RSGs are also rarely mutated in PCa; instead, overexpression and hyperactivation are significant issues driven by factors like the loss of negative regulators, such as HACE1, a ubiquitin ligase for Rac1 [183]. Hyperactivation of Rac1 fuels cell proliferation, survival, and invasion, especially in CRPC, and is linked to AR signaling [184]. Studies indicate that Q61R is a PCa-associated gain-of-function mutation in Rac1 [185], identified in 6% patients with PCa in the TCGA database. Similarly, the mutational frequency of Rac2, Rac3, and RhoG (no RhoG mutations reported in TCGA) are low in PCa, but their overexpression promotes metastasis [82,186].

Like other classical RSGs, Cdc42 is rarely mutated in PCa (no mutations reported in TCGA), but is often overexpressed, which positively correlates with lymph node and visceral metastasis in mCRPC patients. Significantly, the aggressive phenotype of Cdc42-overexpressed PCa tumors is often mediated by the activation of its downstream target Ack1 [187]. RhoJ (L130P) has been reported in PCa patients in the TCGA database (no RhoQ mutations reported in TCGA), but the significance of this mutation has not been reported. Further studies are needed to identify genetic variations in atypical RSGs in PCa.

## 4. Interaction Between Rho Small GTPases and Their Regulators with the Androgen Receptor

### 4.1. Relationship Between Androgen Receptor and Rho Small GTPases

Many classical RSGs mediate AR signaling pathways that promote PCa migration, invasion, and metastasis [23]. In turn, many of these classical RSGs are modulated by interactions with the AR. Some of the interactions are genomic and involve transcriptional regulation, while others operate through strictly non-genomic mechanisms. Only a few of the 20 Rho small GTPases have been studied with respect to their interaction with the AR. We therefore sought to understand any such interaction through correlation between the expression of the AR with that of the RSGs as reported in TCGA data (Table 4). This analysis indicated meaningful positive correlation between AR and only a handful of RSGs—RhoA, Rac1, Cdc42, RhoQ, RhoU, and the RhoBTBs.

#### 4.1.1. Genomic Interaction Between AR and RhoA via SRF and PKN1/PRK1

**Figure 8:**
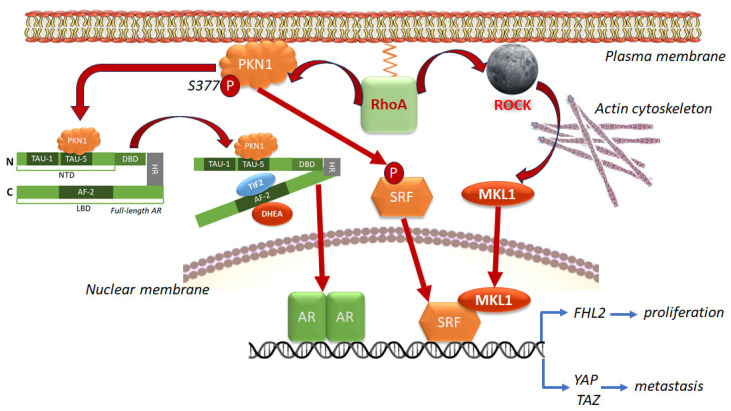
Genomic effects of AR and RhoA on target genes. RhoA influences AR transcriptional activity through multiple pathways. On one hand, RhoA activates ROCK, and this leads to regulation of actin polymerization, which then leads to activation of MKL1/MAL1 and its nuclear translocation. Nuclear MKL1 binds to the transcription factor SRF and promotes its transcriptional activity. On the other hand, RhoA activates the serine/threonine kinase PKN1 which localizes to the membrane when phosphorylated at S377 and interacts with RhoA. Activated PKN1 phosphorylates SRF and also binds to the TAU-5 region on the AR-NTD and enables an AR conformational change, which leads to binding of the AR co-activator TIF-2 to the AF-2 region on the AR-LBD, allowing AR binding to and activation by ligands such as adrenal androgens. This allows AR nuclear translocation and co-regulation of SRF target genes such as *FHL2*, *YAP*, and *TAZ*.

Like Cdc42, mentioned above [100], RhoA also exerts genomic influences on cellular function by regulating SRF, a transcription factor essential for regulating genes involved in actin cytoskeleton dynamic and cellular migration. Studies show that SRF is a mediator of AR activity in PCa [23]. Some of the transcriptional targets of SRF are androgen-responsive, suggesting cooperation between these two factors in the management of their transcription. Significantly, RhoA is an upstream regulator of SRF [188], and mediates the androgen-responsiveness of many of the common targets [23]. Androgen regulation of the SRF target gene *FHL2* requires RhoA. Androgen treatment induces RhoA and ROCK activity, resulting in actin polymerization, which enables nuclear translocation of the SRF cofactor megakaryocytic acute leukemia [MAL, also called megakaryoblastic leukemia 1 (MKL1)] (Figure 8). In turn, SRF is essential for actin cytoskeletal organization and focal adhesion assembly [189].

The RhoA effector protein kinase N1 (PKN1, also known as protein kinase C-related kinase 1 or PRK1) transactivates the SRF and transduces androgen-responsiveness to SRF targets [190]. PKN1 is an important downstream effector of RhoA involved in modulation of AR transcriptional activity. PKN1/PRK1 is usually cytoplasmic, but its phosphorylation at S377 enables its incorporation into the plasma membrane, which is necessary for its interaction with RhoA [191]. Stimulation of PKN1/PRK1 signaling downstream of RhoA results in ligand-dependent AR activation [192]. In the AR-NTD lies the signal-inducible transactivation unit 5 (TAU-5). Upon activation of the RhoA signaling cascade, PKN1/PRK1 transactivates the AR by directly binding to TAU-5 [192]. Transcription Intermediary Factor 2 (TIF-2) is a well-known AR co-activator that interacts with the AR through the activation function 2 (AF-2) region on the AR-LBD [193]. Upon ligand binding, the AR undergoes a conformational change that exposes the AF-2 region to TIF-2 [192]. PKN1/PRK1 binding to TAU-5 promotes the binding of AR with TIF-2, which allows AR transactivation by minor androgens including adrenal androgens in CRPC (Figure 8). Thus, the RhoA/PKN1 pathway is important in promoting CRPC progression.

#### 4.1.2. RhoA and AR Compensate for Each Other’s Functions

In the prostate, activation of RhoA is mostly enhanced by DHT [194]. Androgen-stimulated PCa cell invasion is mediated by RhoA activation [195]. However, in some conditions, RhoA negatively regulates AR function [196]. As a result, when AR transcriptional activity is suppressed, RhoA sometimes compensates for this loss in function. In human patients, tumors that have low AR activity but are highly aggressive (called low-PSA/high-grade tumors) express high RhoA levels [197]. E6-AP, a dual function steroid hormone receptor co-activator and ubiquitin-protein ligase [encoded by the gene ubiquitin-protein ligase E3A (*UBE3A*)], regulates the PI3K/Akt pathway that modulates cell survival in both HSPC and CRPC [198]. In HSPC, E6-AP acts as an AR co-activator to amplify androgen-dependent activation of PI3K-Akt signaling. In contrast, in CRPC, E6-AP modulates PI3K-Akt signaling by regulating the levels of RhoA, a negative regulator of Akt. Thus, AR and RhoA mediated E6-AP’s effects on Akt under alternate conditions. The opposing effects of AR on RhoA was shown in neurons as well, where androgens were shown to induce neuritogenesis in PC12 cells through inactivation of RhoA [199].

#### 4.1.3. Non-Genomic Interaction Between the AR and RhoA/B

Androgens also exert non-genomic effects on RhoA—for example, an albumin-conjugated androgen, testosterone-BSA, so generated so that it can only work at the plasma membrane and not in the nucleus (since it is too big to enter the nucleus), shows significant induction of actin polymerization and apoptosis and triggers RhoA/B and Cdc42 activation in DU145 cells that lack nuclear AR expression but express mAR, indicating a mAR-dependent non-genomic mode of operation [65]. The study indicates that classical RSGs are major AR effectors at the plasma membrane, controlling actin reorganization and apoptosis in PCa. In LNCaP cells, mAR binds testosterone-HSA and induces a rapid and moderate 1.5-fold activation of RhoA [200]. Endostatin, a naturally occurring protein fragment of collagen XVIII that inhibits angiogenesis, downregulates RhoA only in cells that express AR but not in cells that lack AR expression [201]. This interaction is non-geotropic and endostatin regulates only cell migration but not proliferation. These non-genomic effects of AR on RhoA are important in other organs as well. In smooth muscle cells, androgens induce a rapid activation of RhoA and translocation to the plasma membrane [202].

#### 4.1.4. Interaction of the AR with Other Classical Rho Small GTPases

Like RhoA, AR also induces the activation of RhoB [203], and AR variants lacking the LBD upregulate RhoB expression [75]. However, no reports on the interaction between RhoC and AR have been reported so far. Rac1 is also shown to interact with the AR pathway via MAPK signaling [24]. Nuclear localization of AR was enhanced by the addition of geranylgeraniol (GGOH), which geranylgeranylated Rac1 and Cdc42 [204]. In turn, the AR binds to Filamin A (FlnA) and co-localizes at intermediate actin filaments, which regulates AR extranuclear functions, leading to Rac1 activation and consequent cell motility [205,206]. Rac1 is upregulated in enzalutamide-resistant PCa cells, and its knockdown sensitizes PCa cells to this drug [88]. Rac signaling was critical in AR-dependent PCa signaling and Rac1 inhibition synergized with AR inhibitors to prevent AR transcriptional activity [183]. Therefore, Rac1 may play a crucial role in enzalutamide resistance.

Rac1 also was found to participate in non-genomic activation of mARs in PCa, which can modify the actin cytoskeleton and increase PSA levels within minutes [207]. Exposure of LNCaP cells to testosterone-BSA resulted in phosphorylation of FAK, the association of FAK with PI3K, and the subsequent activation of Cdc42/Rac1, which regulate actin organization [207]. AR transactivation is also inhibited by Cdc42 [208]. In this case, a member of the PAK family, PAK6, was activated by AR binding at the AR hinge region, while also bound to GTP-Cdc42, and likely mediates this effect [209]. The rise in AR-low mesenchymal-like CRPC cells are selectively vulnerable to Cdc42-PAK7 inhibition by statins [210]. Thus, similar to RhoA, other classical RSGs also activate AR while the effect of AR on the classical RSGs are more context-dependent. While in some cases, especially when the AR is membrane-bound, AR activates RSGs; in other cases, especially when localized to the nucleus, AR knockdown upregulates the RSGs. It is likely that the two forms of AR (nuclear vs. membranous) work in opposing ways to affect RSG function. Atypical RSGs have not yet been linked to AR functioning at either the membrane or the nucleus, except for cases where studies showed that both the AR and the RSG are affected by the same treatment [126,211].

### 4.2. Linking AR to RSG Dysregulation via Its Regulators

Above, we described the effects of the AR on the RSGs. However, RSG function depends on its regulators—RhoGEF, RhoGAP, and RhoGDI. Therefore, it would be of interest to see whether the AR in PCa is affected by these regulators.

#### 4.2.1. How the AR Controls and Is Controlled by RhoGEFs

The RhoGEF Vav3, a member of the Dbl family of RhoGEFs, is overexpressed in PCa compared to benign prostatic hyperplasia (BPH), a common condition characterized by the enlargement of the prostate gland [212]. Vav3 is also upregulated in CRPC compared to HSPC [143,213] and enhances growth factor activation of AR in the absence of testicular androgen [24]. Growth factor activation of Vav3 enables nuclear localization and ligand-independent activation of AR via Rac1 and MAPK [24]. Vav3 overexpression causes prostatitis and PCa initiation, together with an increase in AR and NF-kB signaling [214]. Inhibition of Vav3 enhances the effects of docetaxel in PCa cells [215] and increases cell death in both HSPC and CRPC [213].

Vav3 has a modular structure including N-terminal Calponin Homology (CH) and Acidic (AC) domains, central Diffuse B-cell lymphoma (Dbl) Homology (DH) and pleckstrin homology (PH) domains, followed by cysteine-rich (C1) and proline-rich (PR) regions and Src Homology (SH2 and SH3) domains at the C-terminal end [216]. The DH and C1 domains are crucial for its GEF activity, while the SH2/SH3 domains bind proline-rich sequences on target proteins to regulate localization and form complexes. Vav3 is expressed in the nucleus as well as in the cytoplasm, and the PH domain is required for its nuclear localization [212,217]. Vav3 nuclear translocation and interaction with AR is stimulated by DHT [218]. Nuclear Vav3 transactivates the AR, whereas membrane targeting of Vav3 abolished this function [217]. The DH domain of Vav3 is necessary for AR activation, whereas the AC and SH2/SH3 domains oppose it [218]. The Vav3 PH domain and the AF1 region of AR-NTD are both necessary for Vav3-induced AR activation [219]. Vav3 and AR are recruited to the same transcriptional complexes [217], indicating its role as an AR co-regulator [212].

Interaction between N- and C-termini of full-length AR is essential for Vav3 stimulation of AR transcriptional activity [217,218]. However, Vav3 can also potentiate AR transcriptional activity in AR variants lacking the AR-LBD [212,219]. Since Vav3 DH domain interacts with the N-terminal region of AR, specifically the TAU-5 region, it can also interact with AR-V7 and other AR variants but disrupts AR-V7 interaction with other AR coactivators such as Src1 and Vav2 [220]. Vav3 likely promotes AR-V7 transportation to the nucleus as Vav3 knockdown resulted in lowered nuclear AR-V7 but did not alter overall AR-V7 expression [212].

While the GEF function of Vav3 is not required for AR activation [217], some publications report that Vav3 did not directly interact with full-length AR [219], suggesting mediation by an RSG. Rac1 is regulated by Vav3, and AR can be transactivated by Rac1 [24]. Some effects of Vav3 were independent of AR ligand binding but were mediated by the PI3K/Akt pathway [213]. It is likely that in these cases, Vav3 activated non-genomic AR activity via PI3K-Akt signaling at the cytoplasm/membrane [218]. These processes are likely to be mediated by Rac1 since the PI3K/Akt pathway is a strong regulator of this RSG [221].

However, Vav3 is not the only RhoGEF that is known to regulate—or be regulated—by the AR. Vav2, which belongs to the same family, and has a structure similar to that of Vav3, has also been implicated in the regulation of the AR [142]. Vav2 overexpression promoted PCa proliferation and metastasis by activating the PAK1/AKT signaling pathway through PAK1 phosphorylation [142]. Further, Vav2 induced enzalutamide resistance in CRPC by enhancing AR/AR-V7 protein stability.

Other GEFs that mediate the effects of RhoA on the AR have also been identified. ARHGEF2, another member of the Dbl family of RhoGEFs, has been identified as a pivotal androgen-repressed gene [222]. *ARHGEF2* encodes GEF-H1, which contains a C1 domain at the N-terminal, followed by an intervening (Inv) domain linking the C1 to the DH and PH domains at the center, and ending with a C-terminal auto-inhibitory domain (CTD) [223]. AR binds to the ARE-containing enhancer of *ARHGEF2* and communicates with the promoter region to repress its expression [222]. AR inhibition relieves the suppression, whereby *ARHGEF2* is activated and promotes CRPC growth and survival. Taken together, the literature so far has pointed to a role for Vav2 and Vav3 in regulating the AR and for the AR to regulate ARHGEF2.

#### 4.2.2. How the AR Controls and Is Controlled by RhoGAPs and RhoGDIs

Only a few studies report on the interaction of AR with the other RSG regulators, RhoGAPs and RhoGDIs. Rho GTPase-activating protein 21 (ARHGAP21), which acts specifically on RhoA, RhoC, and Cdc42 [147], is the only RhoGAP known to interact with the AR [224]. ARHGAP21 silencing in LNCaP prostate cancer cells decreases AR transcriptional levels. In contrast, RhoGDIα is a negative regulator of AR signaling pathway [225]. RhoGDIα interacts with the N-terminal domain of AR, prevents AR nuclear translocation and inhibits the transactivation of AR target genes [225]. It downregulates AR signaling in PCa, prevents AR nuclear translocation and inhibits transactivation of AR target genes [225]. Co-immunoprecipitation assays show that RhoGDIα physically interacts with the N-terminal domain of AR and suppresses PCa growth. The other RhoGDIs, RhoGDIβ and RhoGDIγ, have not been investigated with respect to AR transactivation or AR regulation.

## 5. Conclusions

In this systematic review, we described the interaction between the AR signaling pathway and the Rho family of small GTP-binding proteins. We learn that the AR, which is a ligand-activated steroid nuclear receptor and transcription factor, mediates the initiation and progression of PCa. Apart from regulation of proliferation and survival, the AR also controls metastasis, which is mostly modulated by the Rho small GTPases. These proteins are membrane-bound when active and mediate most of the migratory and invasive functions of tumor cells. The RSGs are molecular switches that are GTP-bound when active and GDP-bound when inactive, which allows them to transmit signals from receptors to downstream pathways via recruitment of effector kinases such as ROCK and PAK. There are multiple RSG subfamilies—including three “classical” subfamilies—Rho, Rac, and Cdc42, which are regulated by the RhoGEFs, RhoGAPs, and RhoGDIs, and five atypical subfamilies—Rif, Rnd, Wrch, RhoH, and RhoBTB, which are either constitutively active or undergo fast intrinsic cycling between the GDP and GTP states. While classical RSGs are mostly oncogenic (except RhoB), the atypical RSGs, with some exceptions, are usually tumor-suppressive in nature.

The AR influences cell migration and invasion partly by regulating classical RSGs in two ways—(i) as a transcription factor in the nucleus, it exerts genomic regulation of RSG expression and that of their regulators; (ii) at the plasma membrane, the AR can have a non-genomic interaction with the RSGs. In turn, the RSGs influence AR function and affect both ligand-bound and ligand-independent AR transcriptional activity, thereby enabling tumor progression. Androgens also interact with RSGs via certain GPCRs at the plasma membrane that bind androgens, even in cells that do not express the classic AR. At present, mPCa is treated with ADT with and without ARSI, including ABI, ENZA, APA, DARO, etc. Since these treatments all involve inhibition of androgen binding to the AR-LBD and preventing subsequent translocation of the AR to the nucleus, they are unable to prevent non-genomic interactions between AR and classical RSGs, which contributes to the resistance to these treatments and progression of mCRPC. In this review, we have identified a number of drugs targeting RSGs that may be developed together with ARSI to prevent mCRPC progression. These drugs are, however, in early stages of development—we identified only one that is currently in Phase I trials, and none are in PCa trials. Taken together, these reports indicate an important role for classical RSGs in promoting tumor metastasis through both genomic and non-genomic mechanisms mediated by nuclear and membrane-localized AR.

## Figures and Tables

**Table 1:** Comparison of the Rho family of small GTPases.

Classification	Rho Subfamily	Subfamily Members #	Molecular Weight (kDa) *	Amino Acids *	Base Length *	Chromosome *	Exons **
Classical	Rho	RhoA	21.8	193	53,860	3p21.31	7
		RhoB	22.1	196	2367	2p24.1	1
		RhoC	22	193	6308	1p13.2	7
	Rac	Rac1	21.5	192	29,441	7p22.1	7
		Rac2	21.4	192	34,325	22q13.1	8
		Rac3	21.4	192	2527	17q25.3	6
		RhoG	21.3	191	13,982	11p15.4	2
	Cdc42	Cdc42	21.3	191	48,734	1p36.12	8
		RhoJ/TCL	23.8	214	89,395	14q23.2	6
		RhoQ/TC10	22.7	205	42,883	2p21	6
Atypical	Rif	RhoD	23.5	210	15,171	11q13.2	4
		RhoF/Rif	23.6	211	25,650	12q24.31	5
	Rnd	Rnd1	26.1	232	8726	12q13.12	5
		Rnd2	25.4	227	6811	17q21.31	7
		Rnd3/RhoE	27.4	244	19,503	2q23.3	6
	Wrch	RhoU/Wrch1	28.2	258	102,023	1q42.13	7
		RhoV/Wrch2/Chp	26.2	236	2021	15q15.1	3
	RhoH	RhoH/TTF	21.3	192	55,957	4p14	13
	RhoBTB	RhoBTB1	79.4	696	141,108	10q21.2	24
		RhoBTB2	82.6	727	69,697	8p21.3	16

* Incorporated from GeneCards^®^—The Human Gene Database. ** Incorporated from NIH National Library of Medicine—National Center for Biochnology Information—Gene database. # Data reported in humans only.

**Table 2:** Major functions and isoprenylation of Rho small GTPases.

Classification	Subfamily	Subfamily Members	Major Function *	Lipidation ^†^
Classical	Rho	RhoA	tumor cell proliferation and metastasis, cytoskeletal organization	GG
		RhoB	mediates apoptosis, tumor suppression, protein trafficking	GG, F, P
		RhoC	assembly of focal adhesions and actin stress fibers	GG
	Rac	Rac1	cell growth, cytoskeletal reorganization, and the activation of protein kinases	GG
		Rac2	secretion, phagocytosis, and cell polarization	GG
		Rac3	cell spreading, actin-based protusions (lamellipodia, membrane ruffles)	GG
		RhoG	lamellipodium formation and cell migration	GG
	Cdc42	Cdc42	actin polymerization, epithelial cell polarization, formation of filopodia	P, GG
		RhoJ/TCL	focal adhesions in endothelial cells may regulate angiogenesis	P, GG
		RhoQ/TC10	sarcomere assembly, epithelial cell polarization, epithelial cell polarization	P, GG
Atypical	Rif	RhoD	endosome dynamics, actin cytoskeleton reorganization, membrane transport	GG
		RhoF/Rif	actin filament organization	GG
	Rnd	Rnd1	response to extracellular growth factors	F
		Rnd2	regulation of neuronal morphology and endosomal trafficking	F
		Rnd3/RhoE	negative regulator of cytoskeletal organization leading to loss of adhesion	F
	Wrch	RhoU/Wrch1	induce filopodium formation and stress fiber dissolution, regulate the actin cytoskeleton, adhesion turnover and increase cell migration, regulation of cell morphology, cytoskeletal organization, and cell proliferation	P
		RhoV/Wrch2/Chp	control of the actin cytoskeleton	P
	RhoH	RhoH/TTF	negative regulator of cell growth and survival	GG
	RhoBTB	RhoBTB1	organization of the actin filament	Nil
		RhoBTB2	candidate tumor suppressor, regulates mitotic cell division	Nil

* Incorporated from GeneCards^®^—The Human Gene Database. ^†^ GG—Geranylgeranylation, F—Farnesylation, P—Palmitoylation (information derived from UniProt^®^ public database).

**Table 3:** Downstream targets of Rho small GTPases.

Classification	Rho Subfamily	Subfamily Members	Downstream Targets	
			Established	Others (Binding Partners) *
Classical	Rho	RhoA	ROCK, Dia, LIMK	ARHGAP1, ARHGAP5, ARHGDIA, ARHGEF11, ARHGEF12, ARHGEF3, CIT, DGKQ, DIAPH1, GEFT, ITPR1, KCNA2, KTN1, MAP3K1, PKN2, PLCG1, Phospholipase D1, protein kinase N1, RAP1GDS1, RICS, TRIO, and TRPC1
		RhoB	PRK1, Sema3A, plexinA4, and Src	NF-κB, ROCK1, and EGFR phosphatase PTPRH
		RhoC	ROCK, Dia, FMNL3	IQGAP1, MMP9, the MAPK pathway, Notch1, and PI3K/AKT
	Rac	Rac1	PAK1, JNK1/2, MLK2/3, PI3K/Akt, NF-kB	Scar/WAVE complex, LIMK/cofilin pathway, and mTOR
		Rac2	p67phox, NOX2, PAK1, Bcl-xL	PI 5-kinase
		Rac3	GIT1, PAK1, PI 5-kinase, E-cadherin	CCND1, MYC, and TFDP1
		RhoG	Rac1, DOCK/ELMO2, Kinectin, MLK3, PLD1, Filamin, SGEF, EphA2	
	Cdc42	Cdc42	PAK, MLK, WASP, IQGAP, PI3K, PAR6, p70S6K, Akt, PIP2, PIP3	CD11b, microtubules, and cell adhesion molecules
		RhoJ/TCL	PAK, ERK, RAC1, MOESIN	PlexinD1 and VEGFR2
		RhoQ/TC10	ROCK, PAK	WASP, RACK1, LIMK, MLCK, MLCP
Atypical	Rif	RhoD	ROCK, mDia, PAK	
		RhoF/Rif	ROCK	Actin binding proteins, cell adhesion and migration proteins, cell polarization proteins, Wnt/b-catenin signaling
	Rnd	Rnd1	ERK, p53, Notch, Plexin	Proteins involved in innate immunity
		Rnd2	pragmin, p38	
		Rnd3/RhoE	ROCK1, PLEKHG5, ARHGAP5, UBXD5, NF-kB/p65	
	Wrch	RhoU/Wrch1	PAK, IQGAP1, Rhotekin	
		RhoV/Wrch2/Chp	PAK1, JNK, AKT, ERK, EGFR	
	RhoH	RhoH/TTF	BCL-6, BLIMP-1, KAISO, ROCK1, PKCα	Inflammatory neutrophil functions
	RhoBTB	RhoBTB1	PDE5, Cullin-3	Actin polymerization, METTL7B, METTL7A
		RhoBTB2	LKB1, HIPPO, CXCL14	

* Only the most prevalent ones are listed.

**Table 4:** Regulation of Rho small GTPases in prostate cancer and its relationship with the androgen receptor. Significant subfamily members are **bolded**.

Subfamily Members	Expression in Prostate Cancer vs. Non-Tumor Tissue ^‡^	Hazard Ratio (Disease Free Survival) **	Correlation with Androgen Receptor Expression ^†^
RhoA	**Increased**	**1.3**	0.48 *
RhoB	Decreased *	**0.85**	0.013
RhoC	**Increased**	1.8 *	0.042
Rac1	**Increased**	**1.1**	0.36 *
Rac2	No change	1.6 *	0.01
Rac3	Increased *	**1.3**	−0.00066
RhoG	No change	1.3	−0.13 *
Cdc42	**Increased**	**1.2**	0.53 *
RhoJ/TCL	Decreased *	1.1	0.12 *
RhoQ/TC10	**Decreased**	**0.84**	0.39 *
RhoD	**Increased**	**1.2**	−0.15 *
RhoF/Rif	Decreased	1.9 *	0.13 *
Rnd1	**No change**	**0.99**	-0.02
Rnd2	Decreased *	**0.75**	−0.2 *
Rnd3/RhoE	Decreased *	**0.76**	-0.029
RhoU/Wrch1	**Increased**	1.5 *	0.27 *
RhoV/Wrch2/Chp	**No change**	**1.1**	−0.074
RhoH/TTF	**Increased**	1.5 *	0.13 *
RhoBTB1	Decreased *	1.4	0.28 *
RhoBTB2	**Decreased**	**0.83**	0.21 *

* Significant change. ^‡^ TCGA tumor (n = 492) vs. normal from TCGA and GTEx combined (n = 152), analyzed by GEPIA2. ** Based on TCGA data—analyzed by GEPIA2. ^†^ Intra-tumor Pearson Correlation Coefficient, based on TCGA data, analyzed by GEPIA2.

**Table 5:** List of drugs affecting Rho small GTPase function mentioned in this paper.

Name of Drug	Target	How It Affects Rho Small GTPases
Y-27632 dihydrochloride	ROCK1/ROCK2, c-myc	inhibits signaling downstream of RhoA, RhoC
CCG-1423	Rho/SRF pathway inhibitor	inhibits transcriptional activity downstream of SRF
Rhosin hydrochloride	RhoA, RhoC	inhibits binding of Rho GTPase with the GEF LARG
NSC126188	RhoB inducer	activates RhoB transcription
RV001	RhoC (vaccination)	immune responses
NSC 23766	Rac1	inhibits Rac1 binding by the Rac-specific GEF Trio or Tiam1
1A-116	Rac1	anti-tumor activity
Z62954982	Rac1	preventing Rac1 from interacting with Tiam1
EHT 1864 2HCl	pan-Rac	directly binding to and inhibiting Rac1, Rac1b, Rac2, and Rac3
EHop-016	Rac1, Rac3	inhibits the association of Vav2 with Rac GTPase
Azathioprine(BW 57-322)	rac1	immunosuppressive drug.
MBQ-167	Rac1/Cdc42	ongoing Phase I clinical trial
ZCL278	Cdc42	targeting Cdc42–intersectin interaction
MLS-573151	Cdc42	blocking the binding of GTP to Cdc42
CID44216842	Cdc42	guanine nucleotide binding inhibitor
ML141 (CID-2950007)	Cdc42, Rac1, Rab2, Rab7	potent, selective, and reversible non-competitive inhibitor of Cdc42 GTPase
ARN25062	RhoJ/Cdc42	inhibits S6 phosphorylation and MAPK activation

## Data Availability

No data was generated for this article.
